# Genome-wide microRNA expression profiling in placentae from frozen-thawed blastocyst transfer

**DOI:** 10.1186/s13148-017-0379-6

**Published:** 2017-08-03

**Authors:** Hitoshi Hiura, Hiromitsu Hattori, Norio Kobayashi, Hiroaki Okae, Hatsune Chiba, Naoko Miyauchi, Akane Kitamura, Hiroyuki Kikuchi, Hiroaki Yoshida, Takahiro Arima

**Affiliations:** 1 Department of Informative Genetics, Tohoku University Graduate School of Medicine, 2–1 Seiryo-cho, Aoba-ku, Sendai, 980-8575 Japan; 2 Laboratory of Animal Reproduction and Development, Graduate School of Agricultural Science, Tohoku University, 1-1 Amamiya-machi, Tsutsumidori, Aoba-ku, Sendai, 981-8555 Japan; 3 Center for Reproductive Medicine, Sendai ART Clinic, 206-13 Nakakecho, Miyagino-ku, Sendai, 983-0864 Japan

**Keywords:** Assisted reproductive technologies (ART), Frozen-thawed embryo transfer (FET), Placenta, MicroRNAs (miRNAs), Microarray, Real-time PCR

## Abstract

**Background:**

Frozen-thawed embryo transfer (FET) is increasingly available for the improvement of the success rate of assisted reproductive technologies other than fresh embryo transfer (ET). There have been numerous findings that FET provides better obstetric and perinatal outcomes. However, the birth weight of infants conceived using FET is heavier than that of those conceived via ET. In addition, some reports have suggested that FET is associated with perinatal diseases such as placenta accreta and pregnancy-induced hypertension (PIH).

**Results:**

In this study, we compared the microRNA (miRNA) expression profiles in term placentae derived from FET, ET, and spontaneous pregnancy (SP). We identified four miRNAs, miR-130a-3p, miR-149-5p, miR-423-5p, and miR-487b-3p, that were significantly downregulated in FET placentae compared with those from SP and ET. We found that DNA methylation of *MEG3*-DMR, not but IG-DMR, was associated with miRNA expression of the *DLK1-DIO3* imprinted domain in the human placenta. In functional analyses, GO terms and signaling pathways related to positive regulation of gene expression, growth, development, cell migration, and type II diabetes mellitus (T2DM) were enriched.

**Conclusions:**

This study supports the hypothesis that the process of FET may increase exposure of epigenome to external influences.

**Electronic supplementary material:**

The online version of this article (doi:10.1186/s13148-017-0379-6) contains supplementary material, which is available to authorized users.

## Background

Recently, due to dramatic increases in success rates with frozen-thawed embryo transfer (FET), obstetric and perinatal outcomes have improved [1, 2]. A large Japanese registry study indicated that FET reduced the incidences of small for gestational age (SGA), low birthweight (LBW), and prematurity when compared with fresh embryo transfer (ET) [3]. In other large studies and comprehensive meta-analyses, FET was associated with increased success rates and decreased obstetric and perinatal complications compared to ET [1, 4, 5]. However, singleton FET is adversely associated with increased risks of macrosomia and large for gestational age (LGA) and higher incidences of placenta accreta and pregnancy-induced hypertension (PIH) [3, 6].

For mice, it has been reported that FET affects the expression of imprinted genes and DNA methylation in both the fetus and placenta [7]. With assisted reproductive technologies (ART), including FET, major epigenetic reprogramming events occur and may expose the epigenome to external influences, preventing proper embryogenesis and placentogenesis [8–10]. In humans, epigenetic alterations of the placenta in utero may increase the risk of developing various diseases later in life such as diabetes mellitus (DM) [11].

MicroRNAs (miRNAs), approximately 22 nucleotides in length, are evolutionarily well-conserved non-coding RNAs that regulate gene expression by binding specific sites of 3′ or 5′ untranslated regions of target genes. They promote target mRNA degradation and translational inhibition, and are considered a mode of epigenetic regulation [12]. Abnormal expression of miRNAs has been observed in pregnancy-associated diseases and recurrent pregnancy loss [13, 14]. Several pregnancy-related miRNAs cluster in the large human genomic regions located in imprinted chromosomes 14q32 (C14MC) and 19q13.42 (C19MC) [15, 16]. Parent-of-origin-specific methylation imprints are established during oogenesis or spermatogenesis and stably maintained through fertilization and subsequent embryonic and placental development in mammals [17].

In this study we, for the first time, performed genome-wide miRNA analysis of term placentae derived from FET, ET, and spontaneous pregnancy (SP). We found that expression of miR-130a-3p, miR-149-5p, miR-423-5p, and miR-487b-3p was decreased in the term placentae derived from FET compared with those derived from ET or SP. Functional analyses suggest that these miRNAs are involved in an increase of neonatal birthweight and potentiality lead to diseases later in life such as DM. This study supports our hypothesis that the process of FET may increase exposure of the epigenome to external influences.

## Methods

### Sample collection

Pregnant women who conceived by SP, FET or ET were recruited from one private hospital. Patient information is shown in Additional file 1: Table S1. There were no severe perinatal complications. Controlled ovarian stimulation was performed with combination of a GnRH agonist, letrozole/GnRH agonist, GnRH antagonist or clomiphene citrate, and recombinant FSH, hMG, and hCG. All inseminations for FET and ET were performed by intracytoplasmic sperm injection (ICSI). After ICSI, embryos were cultured in Quinn’s Advantage Medium at 37 °C for 5 or 6 days in an atmosphere of 6% CO_2_, 5% O_2_, and 89% N_2_ under humidified conditions. Blastocysts were vitrified using a Cryotop (Kitazato BioPharma, Fuji, Japan) and then were immersed and stored in liquid nitrogen (LN_2_) until embryo transfer. To warm vitrified blastocysts, the Cryotop was removed from the LN_2_ and instantly immersed into 3 ml of the medium containing 1.0 M sucrose at 37 °C for 1 min. All patients who underwent FET used the hormone replacement cycle (HRC). Under the HRC, patients were prepared for ET using conjugated estrogens or transdermal estradiol. Placentae were collected from singleton pregnancies after term delivery. Two 1-cm^3^ cubes of the placental attachment of the umbilical cord were excised from term placenta and were washed in PBS at least five times to remove blood cells. For RNA extraction, one was homogenized in 5 ml of Isogen (Nippon Gene, Toyama, Japan) and was preserved immediately. For DNA extraction, the other was immediately transferred into a 1.5 ml tube. All samples were stored at −80 °C until use. We used adjusted birthweight by subtracting the value of the birthweight from the value of the fetal growth curve considering the Japanese gestational age-, gender-, and parity-specific growth standard [18].

### RNA extraction and DNA extraction

Total RNA was isolated from placental tissue using an miRNeasy Mini Kit (Qiagen, Valencia, CA, USA). RNA was quantified using a NanoDrop ND-1000 spectrophotometer (Thermo Fisher Scientific, Waltham, MA, USA). The integrity of the RNA was assessed on an Agilent TapeStation 2200 system (Agilent Technologies, Santa Clara, CA, USA). Purified RNA aliquots were stored at −80 °C until further processing. Genomic DNA was obtained from placental tissue using the standard extraction protocol and stored at −30 °C until use.

### Microarray assay

For microarray analysis, we randomly picked four representative placentae (two male and two female infants) derived from each group. The Agilent SurePrint G3 Human miRNA 8 × 60k (release 21.0) microarray platform for 2588 mature miRNAs was used. Total RNA (100 ng) was labeled and hybridized. The microarray slides were scanned on an Agilent G4900DA SureScan Microarray Scanner System and data were extracted from the scanned images using Agilent Feature Extraction software version 11.0.1.1. Raw microarray data were deposited in the NCBI Gene Expression Omnibus repository (GSE85270). Subsequent analysis was carried out in R (http://www.r-project.org) using functions and packages collected in the Bioconductor project [19]. Raw microarray data were filtered by detection in all samples. Selected miRNA values (*n* = 471) were transformed logarithmically and were normalized by the quantile method.

### Quantitative reverse transcription polymerase chain reaction

Quantitative RT-PCR (qRT-PCR) analysis was performed using TaqMan MicroRNA Assays (Applied Biosystems, Foster City, CA, USA). Briefly, 5 ng of total RNA was used for the synthesis of the first strand cDNA using a TaqMan MicroRNA Reverse Transcription Kit (Applied Biosystems; assay IDs are listed in Additional file 2: Table S2). qRT-PCR was performed on a StepOne Real-time PCR System (Applied Biosystems) using TaqMan Fast Advanced Master Mix (Applied Biosystems). The fold change for miRNA, relative to RNU44 snoRNA, was calculated using the 2^−ΔΔCt^ method [20]. All RT-PCR reactions were performed in duplicate.

### miRNA-predicted target genes and gene ontology (GO) and pathway analyses

Putative target genes of differentially expressed miRNAs were predicted using six available bioinformatics algorithms (miRWalk, DIANA-microT4, miRanda, miRDB, PICTAR2, and TargetScan) with miRWalk2.0 [21]. To reduce the number of false positives, only target genes that were predicted by at least four of the six programs were selected and used for further investigation. The biological annotation and the potential pathway were analyzed using DAVID, version 6.7 [22] and KEGG pathway enrichment analysis [23], respectively.

### Combined bisulfite restriction analysis coupled with the Agilent 2200 TapeStation platform (Bio-COBRA)

Bisulfite treatment of genomic DNA was carried out using an EZ DNA methylation-Gold Kit (Zymo Research, Orange, CA, USA). PCR was performed using TaKaRa EpiTaq HS (Takara Biomedical, Otsu, Japan). The PCR reaction was performed in a volume of 20 μl. Primer and PCR conditions are shown in Additional file 3: Table S3. Of the 20 μl amplicons, 10 μl was digested with 5 units of *Bst*UI, *Hinf*I (New England Biolabs, Hitchin, UK), and *Taq*I (Nippon Gene) for IG-DMR, *MEG3*-DMR, and C19MC-DMR for 16 h, respectively. The digested amplicons were separated and visualized using an Agilent 2200 TapeStation system.

### Statistical analysis

Statistical analyses were performed using R or JMP Pro 11.2.0 (SAS Institute, Cary, NC, USA). Statistical significance among the clinical characteristics, miRNA expression levels quantified by qRT-PCR and DNA methylation levels of the three groups was determined using the Steel-Dwass test at *p* < 0.05. Significance in microarray analysis among the three groups was determined by one-way ANOVA with the Tukey-Kramer post hoc test. Correlations were calculated using the Pearson correlation coefficient. Fisher’s exact test with the Benjamini-Hochberg procedure was used to calculate the *p* values for enriched gene ontology (GO) terms in the DAVID analysis and pathways in the KEGG analysis.

## Results

### Clinical characteristics

We collected 108 placentae, including 28 SP, 64 FET, and 16 ET placentae. The clinical characteristics of these pregnancies are summarized in Table 1. Adjusted birthweight (*p* = 0.0092) and placental weight (*p* = 0.0237) in the FET group were significantly heavier than in the SP group but not the ET group. Mothers were older in the FET (*p* < 0.0001) and ET (*p* = 0.0111) groups than in the SP group. The LGA rate for FET was significantly different (*p* = 0.0150) from SP, but not ET.

**Table 1:** Demographics and clinical characters in this study

	SP	FET	ET
Characteristics	(*n* = 28)	(*n* = 64)	(*n* = 16)
Maternal age (years) (mean ± SD)	30.6 ± 4.1	35.2 ± 3.4	33.9 ± 2.5
Gestational age at delivery (weeks) (mean ± SD)	39.8 ± 0.6	40.0 ± 0.6	39.9 ± 0.8
Parity			
Primiparous (*n*, %)	15 (53.6)	47 (73.4)	13 (81.3)
Multiparous (*n*, %)	13 (46.4)	17 (26.6)	3 (18.8)
Gender of neonate			
Male (*n*, %)	15 (53.6)	36 (56.3)	10 (62.5)
Female (*n*, %)	13 (46.4)	28 (43.8)	6 (37.5)
Birthweight (g) (mean ± SD)	3066.0 ± 302.8	3264.2 ± 360.8	3097.6 ± 376.2
Adjusted birthweight (g) (mean ± SD)	45.9 ± 248.0	239.1 ± 360.3	98.8 ± 352.7
SGA (n, %)	1 (3.6)	3 (4.7)	1 (6.3)
LGA (*n*, %)	1 (3.6)	19 (29.7)	2 (12.5)
Placental weight (g) (mean ± SD)	543.9 ± 69.3	600.6 ± 101.3	583.1 ± 92.0
Obstetrical events			
Cesarean section (*n*, %)	1 (3.6)	14 (21.9)	4 (25.0)
Placenta previa (*n*, %)	0 (0.0)	0 (0.0)	0 (0.0)
Placenta accreta (*n*, %)	0 (0.0)	0 (0.0)	0 (0.0)
PIH (n, %)	0 (0.0)	0 (0.0)	0 (0.0)

*SGA* small for gestational age, *LGA* large for gestational age, *PIH* pregnancy-induced hypertension

### miRNA microarray analysis

**Fig. 1:**
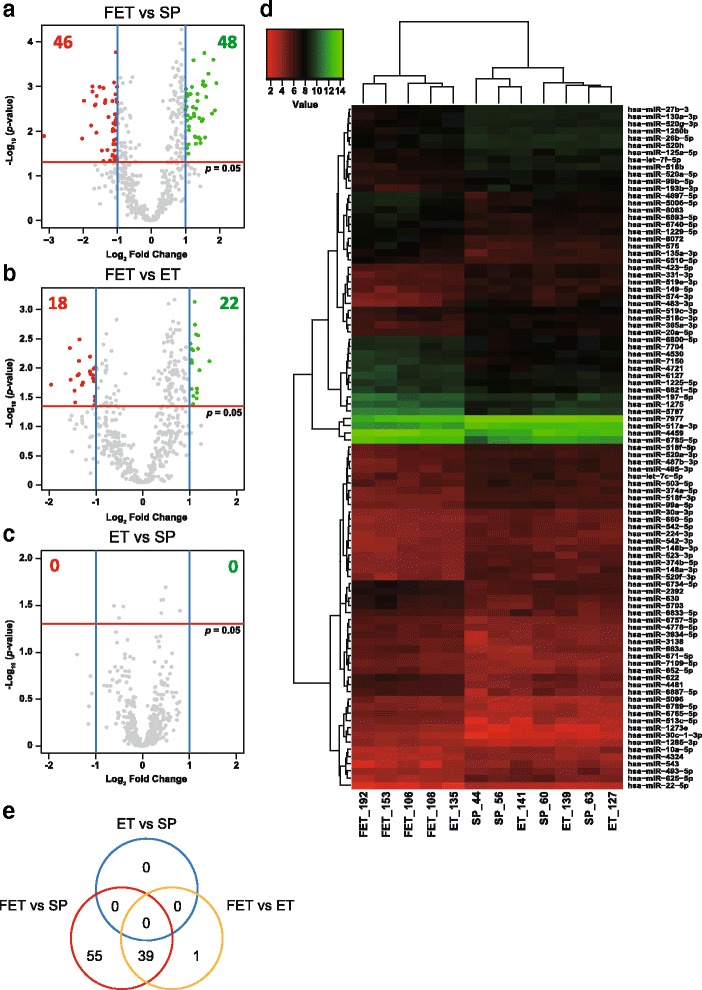
Expression profiles of miRNAs. The volcano plots show miRNA microarrays comparing FET and SP placentae **a**, FET and ET placentae **b** and ET and SP placentae **c**. Plots were constructed using the fold change and *p* values, enabling visualization of the relationship between the fold change and statistical significance. The y-axis is the negative log10 of *p* values (a higher value indicates greater significance) and the x-axis is the difference in expression between two experimental groups as measured in log2 scale. The vertical blue lines correspond to 2-fold up and down, respectively, and the horizontal red line represents a *p* value of 0.05. The green and red points in the plot represent the differentially expressed miRNAs with statistical significance. **d** A heat-map showing differential miRNA expression profiles among the three experimental groups. Red represents downregulated miRNAs and green represents upregulated miRNAs. **e** A Venn diagram showing the number of differentially expressed miRNAs among the three comparisons

To investigate whether placental miRNAs were associated with the characteristics of FET, comprehensive microarray analysis was performed using total RNAs from four placentae each derived from SP, FET, and ET (Fig. 1). Among the 471 human miRNAs detected by the microarray, which targets 2588 mature miRNAs, miRNAs with 2-fold changes between each 2 groups and *p* < 0.05 were identified as differentially expressed. We identified 94 miRNAs significant differentially expressed (46 upregulated and 48 downregulated), between FET and SP placentae, and 40 (18 upregulated and 22 downregulated), between FET and ET placentae. However, we found none between SP and ET placentae. We constructed a heat-map of all differentially expressed miRNAs among the 3 groups and 12 samples separated into 2 groups (4 FETs/1 ET, and 4 SPs/3 ETs) based on the miRNA expression patterns (Fig. 1d). A total of 39 miRNAs, 18 upregulated and 21 downregulated, were commonly differentially expressed in the FET placentae compared with those from SP and ET (Table 2). Notably, among these 95 miRNAs, 18 were located in 3 imprinted regions. Thirteen of them belonged to C19MC, 4 to C14MC, and 1 to the *IGF2* gene (Additional file 4: Table S4).

**Table 2:** Common differentially expressed miRNAs with ≥2-fold changes and *p* < 0.05 in FET placentae

	Fold change				*p* value			
	miRNA	FET vs SP	FET vs ET	ET vs SP	FET vs SP	FET vs ET	ET vs SP	Imprinted domain
Upregulated	hsa-miR-1273e	3.74	2.26	1.65	8.44E-04	5.19E-03	4.09E-01	−
	*hsa-miR-197-5p*	2.13	2.11	1.01	3.20E-02	3.33E-02	1.00E + 00	−
	hsa-miR-30c-1-3p	3.19	2.02	1.58	5.21E-04	4.26E-03	2.97E-01	−
	hsa-miR-4459	2.85	2.31	1.23	5.44E-03	1.21E-02	8.54E-01	−
	hsa-miR-4481	3.00	2.06	1.46	9.52E-04	5.28E-03	4.51E-01	−
	hsa-miR-4530	2.56	2.10	1.22	2.12E-03	5.55E-03	7.78E-01	−
	*hsa-miR-4697-5p*	2.95	2.26	1.30	9.89E-04	3.09E-03	6.83E-01	−
	*hsa-miR-4721*	2.98	2.22	1.34	1.40E-02	3.68E-02	8.09E-01	−
	*hsa-miR-5006-5p*	2.84	2.20	1.29	9.08E-04	2.96E-03	6.61E-01	−
	hsa-miR-513c-5p	2.60	2.23	1.17	1.38E-02	2.49E-02	9.23E-01	−
	hsa-miR-5703	2.71	2.10	1.29	1.87E-02	4.60E-02	8.33E-01	−
	*hsa-miR-575*	3.63	2.67	1.36	3.45E-03	8.49E-03	8.12E-01	−
	hsa-miR-5787	3.43	2.06	1.67	4.06E-03	2.53E-02	4.68E-01	−
	hsa-miR-622	3.05	2.07	1.48	1.72E-03	9.07E-03	4.95E-01	−
	hsa-miR-630	2.69	2.23	1.21	1.50E-02	2.94E-02	9.01E-01	−
	*hsa-miR-6893-5p*	2.49	2.05	1.21	3.14E-03	8.14E-03	7.90E-01	−
	hsa-miR-7150	3.53	2.16	1.64	2.53E-04	2.01E-03	2.69E-01	−
	hsa-miR-8072	2.92	2.15	1.36	1.77E-04	8.19E-04	4.32E-01	−
Downregulated	hsa-miR-10a-5p	0.47	0.46	1.03	9.83E-03	7.11E-03	9.74E-01	−
	*hsa-miR-125a-5p*	0.47	0.46	1.01	1.61E-02	1.41E-02	9.96E-01	−
	*hsa-miR-1260b*	0.46	0.48	0.95	6.70E-03	1.14E-02	9.31E-01	−
	*hsa-miR-130a-3p*	0.46	0.48	0.94	9.96E-03	1.87E-02	9.10E-01	−
	hsa-miR-148b-3p	0.47	0.49	0.97	3.69E-02	4.78E-02	9.85E-01	−
	*hsa-miR-149-5p*	0.41	0.34	1.20	2.66E-02	5.05E-03	5.35E-01	−
	*hsa-miR-193b-3p*	0.25	0.37	0.69	2.08E-03	4.29E-02	1.57E-01	−
	*hsa-miR-224-3p*	0.47	0.49	0.96	2.73E-02	4.02E-02	9.67E-01	−
	*hsa-miR-331-3p*	0.33	0.37	0.89	1.09E-02	2.72E-02	8.23E-01	−
	*hsa-miR-365a-3p*	0.30	0.41	0.74	1.30E-03	2.17E-02	1.78E-01	−
	hsa-miR-374b-5p	0.46	0.49	0.94	1.96E-02	3.44E-02	9.31E-01	−
	*hsa-miR-423-5p*	0.42	0.46	0.91	4.87E-03	1.27E-02	7.98E-01	−
	*hsa-miR-487b-3p*	0.33	0.39	0.85	1.62E-03	8.38E-03	5.01E-01	C14MC
	*hsa-miR-495-3p*	0.35	0.39	0.89	1.02E-03	3.61E-03	6.31E-01	C14MC
	*hsa-miR-518b*	0.32	0.38	0.83	2.65E-03	1.49E-02	4.90E-01	C19MC
	*hsa-miR-518f-3p*	0.38	0.48	0.79	1.08E-03	1.46E-02	2.09E-01	C19MC
	hsa-miR-520a-5p	0.48	0.50	0.97	7.20E-03	1.08E-02	9.60E-01	C19MC
	*hsa-miR-543*	0.36	0.34	1.06	2.72E-02	1.76E-02	9.57E-01	C14MC
	hsa-miR-574-3p	0.24	0.26	0.95	1.48E-02	2.13E-02	9.70E-01	−
	*hsa-miR-7977*	0.32	0.39	0.83	2.51E-03	1.41E-02	4.87E-01	−
	hsa-miR-99a-5p	0.30	0.41	0.73	9.95E-04	1.96E-02	1.45E-01	−

Letters in italics indicate miRNAs confirmed by qRT-PCR in this study

### miRNA expression validation by qRT-PCR

**Fig. 2:**
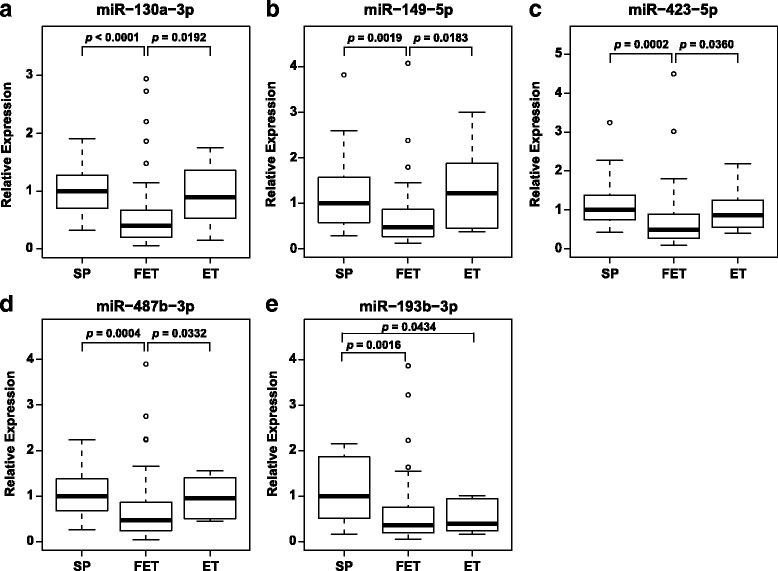
Validation of placental miRNA expression using qPCR. The boxplots show significant downregulation of miR-130a-3p **a**, miR-149-5p **b**, miR-423-5p **c**, miR-487b-3p **d** and miR-193b-3p **e** in the FET placentae. Data were normalized to RNU44 expression and are presented as boxplots with whiskers. The upper and lower limits of the boxes present the 75th and 25th percentiles, respectively. The upper and lower whiskers represent the maximum and minimum values that are no more than 1.5 times the span of the interquartile range (range of the values between the 25th and the 75th percentiles). The circles indicate the outliers. The median is indicated by the line in each box. They were analyzed using the Steel-Dwass test and considered statistically significant when *p* < 0.05

To confirm the microarray results, we conducted qRT-PCR analysis of the expression levels of 21 common differentially expressed miRNAs that had raw signal data with values higher than 50 in all samples of the microarray using 96 placentae. These miRNAs consisted of 6 of the 18 that were upregulated and 15 of the 21 downregulated ones in microarray analysis of the FET placentae. Of these miRNAs, four (miR-130a-3p, miR-149-5p, miR-423-5p, and miR-487b-3p) were significantly downregulated in FET placentae compared with those from SP and ET. miR-193b-3p was significantly downregulated in FET and ET placentae compared with SP placentae. Nine (miR-125a-5p, miR-224-3p, miR-331-3p, miR-365a-3p, miR-495-3p, miR-518b, miR-518f-3p, miR-543, and miR-7977) were significantly downregulated in FET placentae compared with SP, but not ET (Fig. 2 and Additional file 5: Figure S1).

To investigate the relations between miRNA expression and adjusted birthweight and placental weight, we calculated the correlation coefficients of the 21 miRNAs validated by qRT-PCR. There were weak inverse correlations between miR-193b-3p expression and not only placental weight (*R* = −0.29, *p* = 0.0047) but also adjusted birthweight (*R* = −0.29, *p* = 0.0050). For the other 20 miRNAs, there was no significant correlation between miRNA expression and adjusted birthweight or placental weight (Additional file 6: Figure S2 and Additional file 7: Table S5). In four FET placenta-specific miRNAs, there was no significant correlation between miRNA expression and maternal age, whereas there were weak correlations between expression levels of miR-125a-5p, miR-224-3p, miR-331-3p, miR-365a-3p, miR-518b, miR-518f-3p, and miR-543 and maternal age (Additional file 7: Table S5).

### miRNA-predicted target genes and functional analysis

To investigate the pathways in which miRNAs were involved, we searched for potential target genes of the four differentially expressed miRNAs in the FET placentae using six prediction programs, GO terms, and signaling pathways related to the target genes. The target predictions for the four miRNAs showed 4243 putative target sites in 2686 genes. GO enrichment analysis based on the biological process showed that these predicted target genes were clustered into 553 GO terms, among which 161 were significant (corrected *p* value < 0.05) (Additional file 8: Table S6). The top 20 enrichment GO terms are shown in Table 3. Among these, six were associated with positive regulation of transcription, four with the metabolic system, four with the biosynthetic process, and cell migration and cell motion GO terms were enriched. KEGG analysis of these predicted target genes revealed 52 pathways (Additional file 9: Table S7), including 32 significant ones. The top 20 enrichment pathways are shown in Table 4. The signaling, endocrine, gap junction, and focal adhesion pathways, as well as pathways related to some cancers, and the type II DM (T2DM) pathway, were enriched (corrected *p* value = 0.047, Additional file 9: Table S7).

**Table 3:** Top 20 GO terms related to predicted target genes by four miRNAs

GOBPID	Term	Gene count	*p* value	Corrected *p* value
GO:0030182	Neuron differentiation	134	3.59E-18	1.55E-14
GO:0006357	Regulation of transcription from RNA polymerase II promoter	184	5.76E-15	1.24E-11
GO:0048666	Neuron development	103	9.10E-14	1.31E-10
GO:0051254	Positive regulation of RNA metabolic process	130	6.46E-13	6.96E-10
GO:0045893	Positive regulation of transcription, DNA-dependent	129	7.57E-13	6.52E-10
GO:0051173	Positive regulation of nitrogen compound metabolic process	160	2.19E-12	1.58E-09
GO:0045935	Positive regulation of nucleobase, nucleoside, nucleotide and nucleic acid metabolic process	156	2.48E-12	1.53E-09
GO:0007242	Intracellular signaling cascade	268	8.64E-12	4.65E-09
GO:0010557	Positive regulation of macromolecule biosynthetic process	159	1.62E-11	7.74E-09
GO:0009891	Positive regulation of biosynthetic process	166	2.53E-11	1.09E-08
GO:0031328	Positive regulation of cellular biosynthetic process	164	2.73E-11	1.07E-08
GO:0045941	Positive regulation of transcription	141	3.28E-11	1.18E-08
GO:0045944	Positive regulation of transcription from RNA polymerase II promoter	103	3.82E-11	1.27E-08
GO:0010628	Positive regulation of gene expression	144	3.83E-11	1.18E-08
GO:0016477	Cell migration	83	5.15E-11	1.48E-08
GO:0010604	Positive regulation of macromolecule metabolic process	191	3.76E-10	1.01E-07
GO:0006468	Protein amino acid phosphorylation	156	5.48E-10	1.39E-07
GO:0045449	Regulation of transcription	479	8.98E-10	2.15E-07
GO:0006928	Cell motion	118	2.35E-09	5.32E-07
GO:0031175	Neuron projection development	74	4.31E-09	9.29E-07

**Table 4:** Top 20 KEGG pathways related to predicted target genes by four miRNAs

Pathway	Gene count	*p* value	Corrected *p* value
Axon guidance	46	1.73E-08	3.16E-06
Neurotrophin signaling pathway	42	4.05E-07	3.70E-05
MAPK signaling pathway	72	5.70E-07	3.48E-05
GnRH signaling pathway	35	1.15E-06	5.28E-05
Melanogenesis	35	1.51E-06	5.52E-05
Wnt signaling pathway	46	2.94E-06	8.97E-05
Pathways in cancer	79	1.67E-05	4.37E-04
Gap junction	30	2.80E-05	6.41E-04
Pancreatic cancer	26	2.99E-05	6.07E-04
Insulin signaling pathway	40	3.00E-05	5.49E-04
Focal adhesion	53	4.47E-05	7.44E-04
VEGF signaling pathway	26	6.52E-05	9.93E-04
Endocytosis	49	7.02E-05	9.88E-04
Long-term potentiation	24	9.85E-05	1.29E-03
Long-term depression	24	1.27E-04	1.55E-03
ErbB signaling pathway	28	1.35E-04	1.54E-03
Colorectal cancer	27	1.88E-04	2.02E-03
SNARE interactions in vesicular transport	16	2.37E-04	2.41E-03
Phosphatidylinositol signaling system	24	4.08E-04	3.92E-03
Glioma	21	7.09E-04	6.47E-03

### DNA methylation analysis

**Fig. 3:**
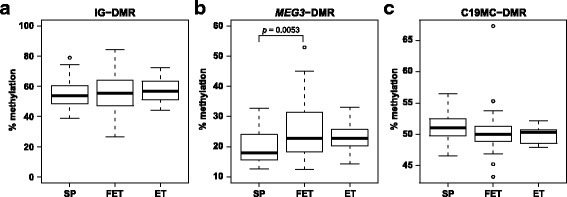
DNA methylation status of differentially methylated regions. The boxplots show the methylation levels of IG-DMR **a**, *MEG3*-DMR **b** and C19MC-DMR **c** in the three groups. Data are presented as boxplots with whiskers. The upper and lower limits of the boxes present the 75th and 25th percentiles, respectively. The upper and lower whiskers represent the maximum and minimum values that are no more than 1.5 times the span of the interquartile range (range of the values between the 25th and the 75th percentiles). The circles indicate the outliers. The median is indicated by the line in each box. They were analyzed using the Steel-Dwass test and were considered statistically significant when *p* < 0.05

We identified 17 differentially expressed miRNAs in imprinted C14MC and C19MC among the three groups (Fig. 1d and Additional file 4: Table S4). To determine whether the downregulation of these miRNAs could be explained by the DNA methylation of imprinted differentially methylated regions (DMRs), we examined the DNA methylation of IG-DMR and *MEG3*-DMR, located in C14MC and C19MC-DMR, using Bio-COBRA (Fig. 3). Although the median methylation levels of IG-DMR and C19MC-DMR were not significantly different among the three groups, that of *MEG3*-DMR in the FET placentae was significantly increased 4.86% (*p* = 0.0053) compared to the SP placentae, but not the ET ones (increased 4.85%, *p* = 0.0979).

## Discussion

With significant improvements of vitrification, the number of FET procedures and success rate have increased in Japan. However, the safety of FET with respect to perinatal complications and the offspring remains unknown. miRNAs are epigenetic mechanisms for the prediction and detection of placenta-mediated pregnancy complications [24]. In this study, we performed genome-wide miRNA analysis comparing term placentae derived from FET, ET, and SP and confirmed four FET placenta-specific miRNAs.

In microarray screening, since the difference between FET and SP was larger than that between FET and SP and there was none between ET and SP, the FET procedure might affect miRNA expression more than ET. The 39 miRNAs commonly differentially expressed in the FET placentae might be involved in conditions such as overgrowth and perinatal complications.

Of the 21 miRNAs that were commonly differentially expressed in the FET placentae compared with those from SP and ET, 14 showed significant differential expression in FET by qRT-PCR. Four of these 14 miRNAs were specifically downregulated in FET. They might contribute to increased birthweight and placental weight; however, their underlying mechanism is unclear. The downregulation of these four miRNAs might have been affected by the FET procedure, not maternal age, although maternal age in the FET and ET groups was higher than for SP and the expression levels of miRNAs decrease with aging [25]. We identified GO terms and pathways related to the four FET-specific miRNAs, as potential causal pathways in the pathogenesis of overgrowth and placenta accreta. Although pathways related to some cancers were enriched, the genes predicted by these four miRNAs might be involved in growth, but not related to cancers themselves. Although there were no differences in the outcomes except for birthweight and placental weight among the three groups, these GO terms and pathways might cause perinatal complications easily.

That the other nine miRNAs showed significantly different expression between FET and SP, but not ET, might be due to the fact that the difference of the miRNA expression levels between FET and SP was greater than between ET and SP. Seven of the nine miRNAs that showed significantly different expression only between FET and SP might be affected by maternal age rather than the FET procedure. The other two might be involved in overgrowth, although they tended to be decreased in FET compared with ET and showed no significant difference.

miR-193b-3p was decreased in FET and ET placentae compared to those from SP and was related to overgrowth. The decreased expression of miR-193b-3p might be due to procedures common to FET and ET, including ovulation-induction methods, insemination, culture system, and embryo transfer. The lack of a significant difference in birthweight and placental weight between ET and SP might be due to factors that compensate for the influence of miR-193b-3p and complement the increases of fetal and placental growth in ET. For 20 miRNAs, each miRNA alone might not affect overgrowth in FET, nevertheless overgrowth might be a synergistic effect of a miRNAs combination. In fact, miRNA binding is partially complementary to multiple mRNA sequences and some miRNAs downregulate large numbers of target mRNAs [26]. In other words, expression of one mRNA is regulated by multiple miRNAs. Therefore, it seems that miRNAs and mRNAs form a complex gene expression network. Moreover, though all human protein-coding genes might not be regulated by miRNAs, more than 30% of human protein-coding genes contain at least one miRNA-binding site [27]. For these reasons, there might be no good correlation between the expression levels of the four miRNAs and the birthweight and placental weight in this study.

In microarray analysis, 13 detectable miRNAs of C19MC and 4 of C14MC were decreased in the FET placentae. Since some C19MC and C14MC miRNAs are associated with pregnancy-related complications [16], the miRNAs we detected might also be. The methylation in *MEG3*-DMR was increased in FET placentae and might play a role in the regulation of C14MC miRNA expression. On the other hand, the methylation of C19MC miRNAs did not differ among the groups and other mechanisms such as histone modifications or unknown promoters might regulate C19MC miRNAs. The methylation of *MEG3*-DMR was increased in FET and ET placentae compared to those from SP, but the lack of a significant difference between ET and SP suggested that the effect for not only miRNA expression but also DNA methylation of FET might be greater than for ET. Many studies have suggested that there is an increased incidence of rare imprinting disorders, including Beckwith–Wiedemann syndrome (BWS) [28] and Silver-Russell syndrome (SRS) [29], associated with ART [30]. *MEG3*-DMR, which is the location of the *MEG3* promoter, encodes large non-coding RNA, as well as *H19*-DMR and *KCNQ1OT1*-DMR involved in SRS and BWS, respectively, might be easily affected by ART. *Meg3* is involved in growth and development although *Meg3*-mutant mice exhibit very unique inheritance [31]. Those results and ours may suggest that some imprinted genes or domains are labile and readily changeable.

In animal studies, the in vitro culture methods and compositions of culture media might induce male large offspring syndrome in ruminants [32, 33]. The effects of ART, including in vitro culture, can be maintained, or more pronounced even after the freezing and thawing of embryos, as embryo vitrification aggravated the loss of methylation of *H19*-DMR in an IVF group with vitrification in mice [7]. Both ovulation-induction treatment and embryo culture increase the perturbation of genomic imprinting [34, 35]. Recently, a randomized study showed that different media for culture of human in vitro fertilization (IVF) embryos affect the birthweights of newborns [36, 37]. The developmental origins of health and disease paradigm postulate that suboptimal growth early in life can program changes that have a lifelong effect on health, increasing the risks for various diseases [38, 39]. Interestingly, not only LBW but also high birthweight infants are at increased risk for future development of type 2 diabetes mellitus (T2DM) [40]. We found a decrease of expression of placental miR-487b-3p, which is located in C14MC, and the pathway associated with T2DM. Recently, islets [41]. Abnormalities occurring in the placenta such as placental insufficiency, placental infarction, and placental angiogenesis might indirectly have adverse effects on the fetus [42–44]. Importantly, the role of environmental epigenetics in FET suggests the possibility of leading to diseases later in life such as DM [45].

There are some limitations to this study. First, this was a study of only Japanese term placentae with no involvement of severe perinatal complications. Second, we could collect only 16 ET placentae. Third, we performed genome-wide miRNA assay comparing three groups. Although the microarray technologies for high-throughput analysis are well established, they cannot detect unknown miRNAs. However, a strength of this study is that both patients with FET and fresh ET underwent the same ICSI protocol at one ART institute in spite of the different backgrounds of the patients.

## Conclusions

In conclusion, our miRNA data support the hypothesis that major epigenetic events taking place during early embryogenesis and the process of FET may increase exposure of the epigenome to external influences. This could lead to being disproportionately large at birth and placenta-mediated pregnancy complications linked to perinatal and adult diseases. Our knowledge that the epigenomes of gametes and newly fertilized embryos are susceptible to environmentally induced epigenetic changes has particularly important implications as changes in lifestyle and modes of reproduction may have long-term implications for human health that are not yet fully appreciated.

## Additional files

Additional file 1: Table S1. Patients’ information. Blastocysts were graded using a blastocyst scoring system [46]. *FSH* follicular stimulating hormone, *HMG* human menopausal gonadotropin. (XLS 51 kb)

Additional file 2: Table S2. List of TaqMan probe ID numbers. (XLS 29 kb)

Additional file 3: Table S3. Primer sequences and PCR conditions in this study. (XLS 29 kb)

Additional file 4: Table S4. Differentially expressed miRNAs with ≥2-fold changes and *p* < 0.05 among the three groups. (XLS 45 kb)

Additional file 5: Fig. S1. Validation of placental miRNAs expression using qRT-PCR. The boxplots show the expression levels of miR-197-5p (a), miR-4697-5p (b), miR-4721 (c), miR-5006-5p (d), miR-575 (e), miR-6893-5p (f), miR-125a-5p (g), miR-1260b (h), miR-224-3p (i), miR-331-3p (j), miR-365a-3p (k), miR-495-3p (l), miR-518b (m), miR-518f-3p (n), miR-543 (o) and miR-7977 (p). Data were normalized to RNU44 expression and are presented as boxplots with whiskers. The upper and lower limits of the boxes present the 75th and 25th percentiles, respectively. The upper and lower whiskers represent the maximum and minimum values that are no more than 1.5 times the span of the interquartile range (range of the values between the 25th and the 75th percentiles). The circles indicate the outliers. The median is indicated by the line in each box. They were analyzed using the Steel-Dwass test and considered statistically significant when *p* < 0.05. (PDF 1684 kb)

Additional file 6: Fig. S2. Correlation between miR-193b-3p expression and adjusted birthweight or placental weight. (a) The miR-193b-3p expression level in the placenta was correlated with adjusted birthweight. (b) The miR-193b-3p expression level in the placenta was correlated with placental weight. Black circles, white circles, and triangles indicate SP, FET and ET samples, respectively. (PDF 893 kb)

Additional file 7: Table S5. Pearson correlation coefficients between miRNA expression and adjusted birthweight, placental weight or maternal age. (XLS 33 kb)

Additional file 8: Table S6. GO term enrichment analysis of target genes. (XLS 272 kb)

Additional file 9: Table S7. KEGG enrichment analysis of target genes. (XLS 45 kb)

## Abbreviations

ART: Assisted reproductive technology
BWS: Beckwith–Wiedemann syndrome
C14MC: Chromosome 14 microRNA cluster
C19MC: Chromosome 19 microRNA cluster
COBRA: Combined bisulfite restriction analysis
DAVID: Database for Annotation, Visualization and Integrated Discovery
DM: Diabetes mellitus
ET: Embryo transfer
FET: Frozen-thawed embryo transfer
FSH: Follicular stimulating hormone
GnRH: Gonadotropin-releasing hormone
GO: Gene ontology
hCG: Human chorionic gonadotropin
hMG: Human menopausal gonadotropin
HRC: Hormone replacement cycle
ICSI: Intracytoplasmic sperm injection
IVF: In vitro fertilization
KEGG: Kyoto Encyclopedia of Genes and Genomes
LBW: Low birthweight
LGA: Large for gestational age
LN_2_: Liquid nitrogen
MAPK: Mitogen-activated protein kinase
PIH: Pregnancy-induced hypertension
rFSH: Recombinant follicular stimulating hormone
RT-PCR: Reverse transcription polymerase chain reaction
SD: Standard deviation
SGA: Small for gestational age
SP: Spontaneous pregnancy
SRS: Silver-Russell syndrome
T2DM: Type 2 diabetes mellitus
VEGF: Vascular endothelial growth factor
