# Supplementary figures and images for: Genome-wide microRNA expression profiling in placentae from frozen-thawed blastocyst transfer
Source: Clin Epigenetics. 2017 Aug 3;9:79. doi: 10.1186/s13148-017-0379-6 (PMC5543431; doi:10.1186/s13148-017-0379-6)

Figure S1-1

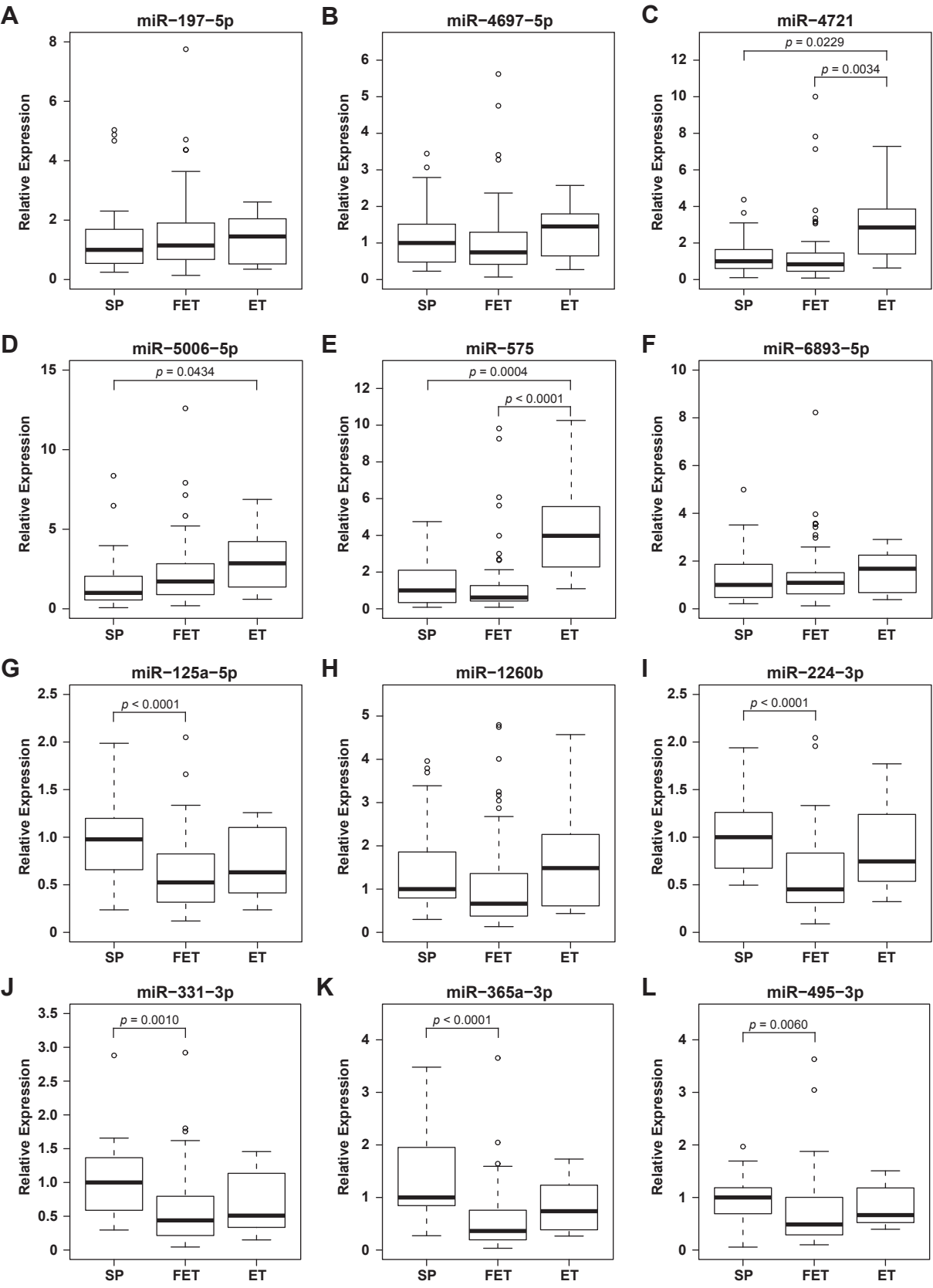

Figure S1-2

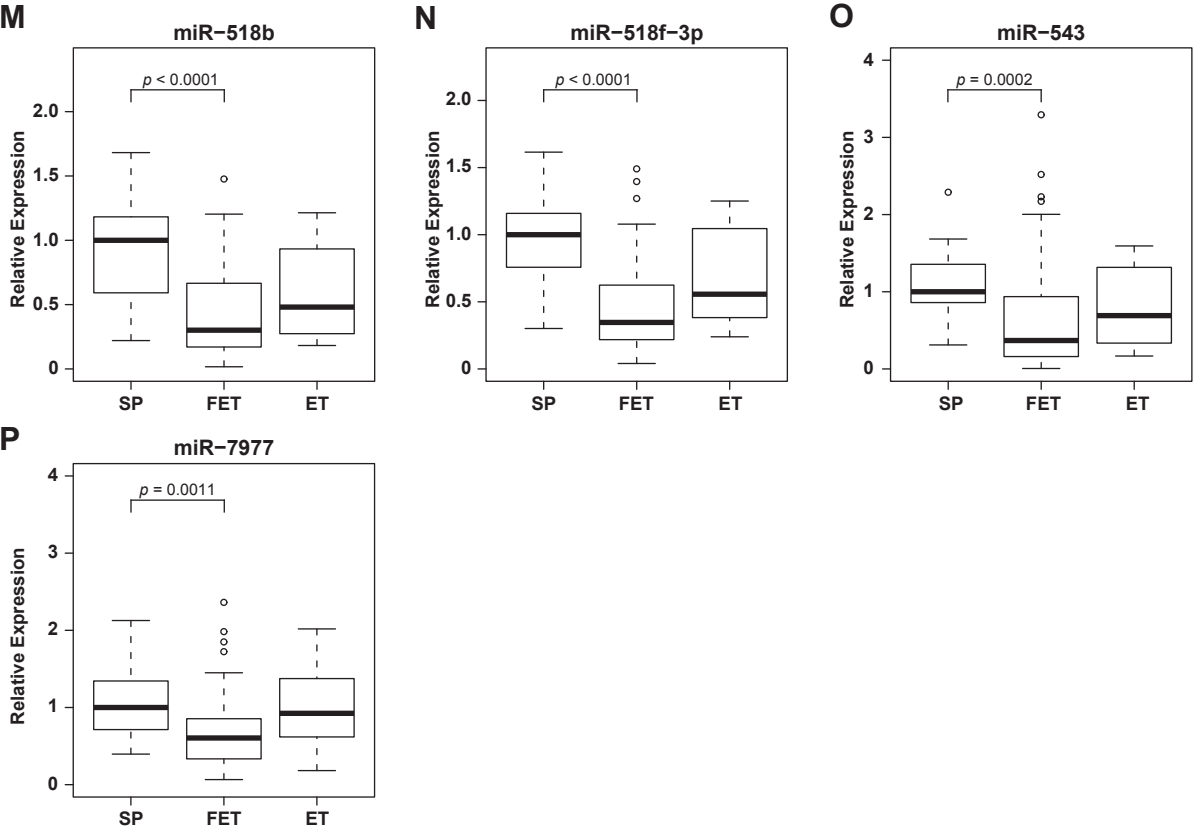

Supplement: Supplementary file 5 — Validation of placental miRNAs expression using qRT-PCR. The boxplots show the expression levels of miR-197-5p (a), miR-4697-5p (b), miR-4721 (c), miR-5006-5p (d), miR-575 (e), miR-6893-5p (f), miR-125a-5p (g), miR-1260b (h), miR-224-3p (i), miR-331-3p (j), miR-365a-3p (k), miR-495-3p (l), miR-518b (m), miR-518f-3p (n), miR-543 (o) and miR-7977 (p). Data were normalized to RNU44 expression and are presented as boxplots with whiskers. The upper and lower limits of the boxes present the 75th and 25th percentiles, respectively. The upper and lower whiskers represent the maximum and minimum values that are no more than 1.5 times the span of the interquartile range (range of the values between the 25th and the 75th percentiles). The circles indicate the outliers. The median is indicated by the line in each box. They were analyzed using the Steel-Dwass test and considered statistically significant when p < 0.05. (PDF 1684 kb) [file 13148_2017_379_MOESM5_ESM.pdf]

Figure S2

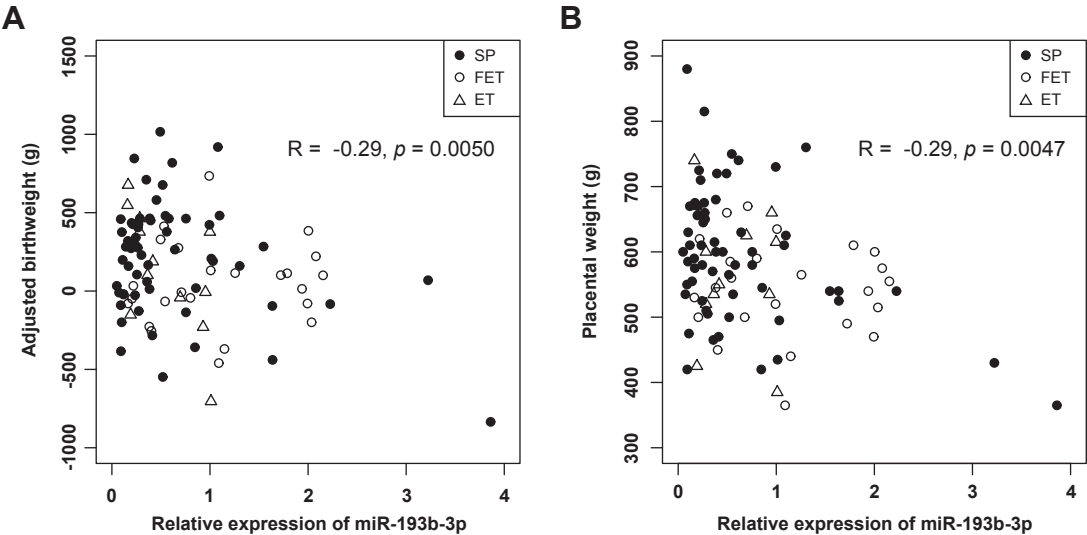

Supplement: Supplementary file 6 — Correlation between miR-193b-3p expression and adjusted birthweight or placental weight. (a) The miR-193b-3p expression level in the placenta was correlated with adjusted birthweight. (b) The miR-193b-3p expression level in the placenta was correlated with placental weight. Black circles, white circles, and triangles indicate SP, FET and ET samples, respectively. (PDF 893 kb) [file 13148_2017_379_MOESM6_ESM.pdf]
